# Deregulation of Cell Death in Cancer: Recent Highlights

**DOI:** 10.3390/cancers12123517

**Published:** 2020-11-26

**Authors:** Vincenzo Carafa, Lucia Altucci

**Affiliations:** Department of Precision Medicine, Università degli Studi della Campania “Luigi Vanvitelli”, 80138 Naples, Italy

The aim of this Special Issue on the deregulation of cell death in cancer is to bring together recent perspectives on the relationship between tumorigenesis and programmed cell death (PCD). According to the World Health Organization, cancer is the second leading cause of death worldwide, and in 2018 it was responsible for an estimated 9.6 million deaths. The human genome is exposed to mutations during the course of life, and while many have no impact on individual health, others (heritable or acquired) are responsible for carcinogenesis. It is becoming ever clearer that defects in intracellular signaling pathways lead to altered gene functions and malignant cellular transformation [1]. Chemotherapy remains one of the most effective forms of cancer treatment. However, the increasing onset of cancer resistance has prompted major scientific efforts to focus on developing novel drugs or alternative chemotherapeutic protocols in order to design new or synergistic anticancer approaches [2,3]. The alteration of cell death mechanisms is a common feature in cancer, and can lead to drug resistance and, consequently, treatment failure. Cell death is a tightly controlled mechanism based on molecular programs in which several genes and related proteins are regulated. The evasion of this cellular mechanism is one of the hallmarks of cancer [4], greatly strengthening the proposition that targeting PCD in tumorigenesis may represent a strategic tool in oncology. In all multicellular organisms, cells are well organized and their life and death are part of the natural cycle. During growth, cells undergo a high rate of proliferation, both to increase in number and to complete the functionality of all components of the organism. At the end of their life cycle, when they have ceased to perform their physiological functions and are no longer required, cells die via the activation of different intracellular processes. The correct dynamic balance between life and death processes, necessary for the production of “new” and the elimination of “old” cells, is fundamental for homeostasis. Following cell injury resulting from exposure to chemical, biological, or physical agents or after a mechanical insult, cells die through a completely unprogrammed accidental cell death modality with consequent cell swelling, membrane rupture, and the activation of inflammation processes [5]. This passive cell death mechanism is named necrosis. In contrast, regulated cell death (RCD) [5], activated upon specific cellular cues and mediated by a family of proteases, is characterized by cell shrinkage, chromatin condensation, nuclear fragmentation, and plasma membrane blebbing [6]. With the accumulation of apoptotic bodies and their subsequent elimination, RCD is classically viewed as a clean, controlled, and noninflammatory process. When RCD occurs in a physiological state as a consequence of the development and/or upon regulation of cell homeostasis, this is known as PCD. The most well-known and best-characterized form of PCD is apoptosis [7]. In the past, cell death was classified based only on morphological criteria and thus included: (i) apoptosis, PCD type I; (ii) autophagy, PCD type II; and (iii) necrosis, lacking PCD type I and II features. Following advancements in biochemical approaches, the Nomenclature Committee on Cell Death devised specific guidelines for a new classification of cell death [8]. As well as evaluating morphological and specific features, these new criteria consider functional aspects, enzyme recruitment, and immunological responses. By taking into account all these perspectives, each cell death process identified was provided with a specific name and description, thus dividing RCD into twelve different classes. While the molecular mechanisms underlying intrinsic and extrinsic apoptosis, autophagy-dependent cell death, necroptosis, pyroptosis, and ferroptosis are already clear, the remaining modalities, including entotic cell death, lysosome-dependent cell death, NETotic cell death, parthanatos, immunogenic cell death, and mitochondrial permeability transition (MPT)-driven necrosis require further investigation [8]. The induction of cell death after chemo/radiotherapy and consequent tumor regression indicate the involvement of apoptosis in tumorigenesis [9]. The expression of anti-apoptotic factors or the inactivation of pro-apoptotic proteins [10] may lead to uncontrolled tumor cell initiation and proliferation, with characteristics of a malignant phenotype. Several cancer types frequently exhibit mutations or the abnormal expression of numerous genes regulating intrinsic or extrinsic cell death pathways and the overexpression of several anti-apoptotic proteins. The direct use of caspase modulators to prevent or induce cell death [11] initially provided encouraging results, but this approach ultimately failed to live up to its promise due to their poor specificity and redundant function in different cell pathways. Consequently, regulating cell fate via alternative modes of cell death, whose components differ from those of apoptotic pathways, represents an attractive new therapeutic approach. One of the re-classified cell death pathways [8] is necroptosis, a well-described form of regulated necrotic cell death [12]; necroptosis mediators belong to both the apoptotic and necrotic pathways [13,14]. Targeting necroptosis may thus be one of several alternative strategies aimed at inducing cancer cell death [15]. Since the necroptotic pathway involves different mediators to those activated in apoptosis, resistant cancer cells may be more sensitive to such an approach [16]. However, the role of necroptosis in carcinogenesis has only recently begun to be investigated, and findings are contradictory. Several reports describe its role in tumor suppression, [1,17], whereas other studies propose the necroptosis-mediated activation of oncogenesis through the release of factors involved in inflammation [18]. Other forms of controlled necrosis, such as MPT and parthanatos, were also associated with cancer development [19] and are, respectively, characterized by the involvement of mitochondrial dysfunction, caused by a rapid increase in inner mitochondrial membrane permeability with the opening of the MTP pore, and hyperactivation of poly (ADP-ribose) polymerase (PARP) [20,21]. The two remaining cell death mechanisms, pyroptosis and ferroptosis, might also play a role in carcinogenesis. Pyroptosis is an inflammatory caspase-dependent form of cell death, while ferroptosis is characterized by lipid peroxide accumulation and iron dependency [22]. Although the molecular mechanisms underlying these cell death modalities are still under investigation, several findings show that the modulation of these molecular processes can inhibit the proliferation and migration of cancer cells [23,24]. In this Special Issue on cell death mechanisms and carcinogenesis, the functional role of proteostasis is also discussed. The proteostasis network is used in the cell system to maintain protein homeostasis [25]. The failure of this process might be harmful to cells and lead to cell death. The induction of proteotoxic stress through the use of small molecules may be of therapeutic interest, particularly in terms of its antitumor activity [26]. However, all potentially druggable proteins or mechanisms involved in cell death pathways may be considered very attractive targets for anticancer therapy. It was recently suggested that neutrophil gelatinase-associated lipocalin (NGAL) has a critical role in cancer biology, sparking interest in its potential as an early diagnostic and prognostic marker for many solid tumors [27]. In this Special Issue, the role of NGAL in chronic lymphocytic leukemia (CLL) is explored, providing evidence that it may be considered a diagnostic or predictive marker in untreated and remission CLL patients, respectively. A comprehensive analysis stressed the involvement of NGAL in the mechanisms of resistance to apoptosis observed in many CLL cells [28]. High anticancer potential was observed using a synthetic drug, the cationic amphiphilic–helical peptidomimetic B18L [29]. By binding BST-2, a key regulator in promoting cancer, B18L was able to induce cell death in drug-resistant and drug-sensitive cancer cells, with low toxicity in normal cells. This study highlights the promising therapeutic activity of B18L, a BST-2-based peptide, for the treatment of breast cancer.

The reactivation of altered or silenced cell death pathways may improve outcomes in cancer. The development of new drugs able to modulate specific molecular targets involved in cell death mechanisms may provide more efficient therapeutic strategies for cancer treatment.
